# A decline in avian cytokine expression with age revealed by commercially available cytokine array

**DOI:** 10.3389/fphys.2023.1171782

**Published:** 2023-04-12

**Authors:** Guy Levkovich, Dana Almagor, Ganesan Saibaba, Inna Bendikov-Bar, Mark Rusal, Dmitri Lokshtanov, Dmitri Shinder, Dror Sagi

**Affiliations:** ^1^ Agricultural Research Organization, Volcani Center, Institute of Animal Science, Rishon LeZion, Israel; ^2^ Koret School of Veterinary Medicine, Hebrew University of Jerusalem, Jerusalem, Israel

**Keywords:** hens, aging, immunology, array, cytokines

## Abstract

Cytokines are secreted immunomodulators that are key regulators of the avian immune response. Currently, the most commonly used method to follow cytokine expression is qPCR, which measures cellular levels of mRNA, rather their extracellular circulating levels. Here we present a commercially available cytokine array designed to assay circulating expression levels of multiple cytokines and immunomodulators simultaneously. Upon minor modifications to the manufacturer protocol, background noise was reduced, leading to a significant increase in the sensitivity of the device. Our data indicate that the array is reliable and produce consistent data between biological repeats. We tested the reproducibility of the array in a biologically relevant context by assessing age-related changes in circulating cytokines. While individual features did not show a consistent pattern, our data revealed a consistent decline in the median of all cytokine values, supporting the validity of the array in studying biological processes.

## Introduction

The immune system of laying hens plays a crucial role in maintaining health and productivity (Schmucker et al., 2021). Unlike laboratory model animals (Bendikov-Bar et al., 2021), the immune system of hens is constantly exposed to various challenges of the complex housing environment of chicken facilities, including pathogens and stress. This makes the laying hen a valuable model for studying immunology in a realistic setting that is similar to humans.

Cytokines are small proteins, 5–25 kDa, that play a crucial role in the regulation of the immune system. They are secreted and recognized mainly by immune cells, serving as the messengers that regulate the maturation, growth, and responsiveness of particular cell populations. Cytokines are important in health and disease, specifically in host immune responses to infection, inflammation, and cancer, and therefore can serve as biomarkers for assessing immune function (Zhang and An, 2007; Tisoncik et al., 2012).

In avian immunology, the most commonly used tool for cytokine detection and quantification is quantitative polymerase chain reaction (qPCR) (Borowska et al., 2019). Quantitative PCR allows the screening of hundreds of targets in parallel for mRNA expression with high-throughput tools, such as 96.96 Dynamic Array (Spurgeon et al., 2008), facilitating rapid and cost-effective analysis of immune responses (Borowska et al., 2019; Xu et al., 2019).

However, this method only measures cellular cytokine expression at the mRNA level and does not provide information about actual concentrations of circulating cytokines (Krzysica et al., 2022).

To address this limitation, there has been increasing interest in directly measuring cytokine expression using antibodies, to improve our understanding of the immune system in different tissues and extracellular environments (Mahfuz et al., 2018; Mucksová et al., 2018; Yan et al., 2019). However, these studies used enzyme-linked immunosorbent assay (ELISA), an assay limited to detecting only one feature at a time. The same limitation also applies to the well-established Western blot assay, which is also less reliable in obtaining quantitative data compared to ELISA (Lu et al., 2023).

One tool for measuring the expression of multiple cytokines in one device is a cytokine antibody array (Huang, 2004; Huang et al., 2021). It is a slide-based device that contains surface-bound antibodies against multiple cytokines, allowing for the simultaneous detection of multiple features in a single well.

Indeed, in humans and mice, cytokine arrays are being used to measure dozens of features simultaneously in a single well (Wilson et al., 2015).

In this study, we evaluated the G-Series Chicken Cytokine array (RayBiotech), a slide-based device designed to identify the expression of nine immunomodulators in chicken plasma.

We found the array to be consistent in replicating experimental data as well as its response to lipopolysaccharide (LPS), a well-established immune stimulator (Simpson and Trent, 2019).

The reliability and reproducibility of the array were further tested in a biologically relevant context by evaluating changes in cytokine expression associated with aging. Our data consistently demonstrated an overall decrease in cytokine expression with age, supporting the validity of the array in studying biological processes.

## Materials and methods

### Animal husbandry

White Leghorn (Lohman) laying hens, all females, were purchased from commercial husbandries (Hasolelim, Israel) at the age of 1 day and raised in the poultry farm of the Volcani Center, Israel. Maintenance conditions and feeding formulas were according to the Lohman guidelines (https://hylinena.com/wp-content/uploads/ 2019/10/Lohmann_LSL-Lite16-2. pdf), with free accesses to food and water.

Throughout the study hens were accommodated in Individual cages (40 × 40 × 43 cm), one hen per cage, to allow longitudinal tracking.

### Blood sampling

From each layer, 1 mL of blood samples was taken from the wing vein and immediately added to a solution of 100 μL heparin-PBS (Sigma) at 10 mg/mL to prevent clotting. Samples were kept on ice for about 1 h and then centrifuged for 20 min at 10,000 rpm in a tabletop cold centrifuge. Plasma samples were aspirated to new tubes (Eppendorf 2 mL, safe lock 0030120094), froze immediately in liquid nitrogen, and stored at −80°C.

### Cytokine array assay

The details of the assay are described in the Result section.

To obtain expression values, the device scanned with a GenePix 4000B scanner (Axon Instruments, GenePix version 5.0) and analyzed with the Raybiotech analysis tool, a data analysis program based on Microsoft Excel technology specifically designed to analyze Raybiotech Antibody Array G Series. Signals were normalized using internal, positive and negative controls included on the array.

### Raw expression data

The raw data extracted from all arrays in this study are summarized in Supplementary file 1. The data are organized and presented according to the various study cohorts.

### Calculation of significance for old-to-young ratio

Significance for old-to-young-ratio were calculated using random permutation of the samples for each of the four sets of array separately. Each array comparing old-to-young hens consisted of 16 samples, eight for each age group. The expression of each feature was calculated as the average over eight samples (animals) of the corresponding age. Then, the median of all feature-averages was obtained. Therefore, each permutation of the 16 samples resulted in a new expression pattern of the array. We then calculated the probability to have the measured median ratio or lower for each array separately, and obtained 0.067, 0.086, 0.076, and 0.2. We therefore multiplied all 4 values to have the significance of all four sets exhibiting lower expression values of the medians with age.

## Results

### An improved washing protocol to reduce signal-to-noise ratio

Signal-to-noise ratio is one of the main limiting factors in obtaining quality data in antibody arrays (Brase et al., 2010; Wellhausen and Seitz, 2012).

Here we present an optimize protocol for cytokine expression in chicken sera. The cytokine profiles were analyzed with a semiquantitative chicken cytokine antibody array that enables simultaneous detection of nine immunomodulators in one well (RayBio G-Series Chicken Cytokine Array 1, Raybiotech, Norcross GA USA https://www.raybiotech.com/chicken-cytokine-array-gs1-en, October 2021).

The slides are made of glass with an array that uses matched pairs of cytokine-specific antibodies for detection, printed on the slides, similar to sandwich-based ELISA. The slides provide a quadruplicate of each specific target cytokine antibody.

The array glass slides were pretreated in accordance with the manufacturer’s instructions with slight modifications, as described below.

Each well in the array was loaded for incubation in several stages, in which we implemented some modifications: first, each well was loaded with 100 μL of sample diluent buffer for 30 min in room temperature; second, each well was loaded with 50 μL of plasma, after 3 s of inducing vortex in the plasma tubes to homogenize the plasma, a step which was not included in the standard protocol, and 50 μL sample diluent buffer, sealed with adhesive film and incubated over-night (approximately 18 h) at 4°C, gently shaken; third, each well was loaded 80 μL Biotin conjugated anti-cytokine mix and incubated for two hours in room temperature; and fourth, each well was loaded with 80 μL Cy3 equivalent dye-conjugated streptavidin and incubated for one hour in room temperature under aluminum foil to avoid exposure to light. After washing, the array was peeled off the slide and the slides were washed under tap with Double-distilled H_2_O to remove any residuals on the slides and were further washed and dried in a centrifuge for three minutes at a speed of 1,000 rpm at 4°C, as an addition to the manufacturer’s protocol in order to avoid water stains on the slides. The slides were then stored under aluminum foil at 4°C.

At the beginning and between stages the slides were washed and dried in accordance with the manufacturer’s manual with an additional modification of emptying the wells between stages by vacuum suction, instead of simple decanting. This step improves the clarity of the slide by efficiently removing residual liquids.

**FIGURE 1:**
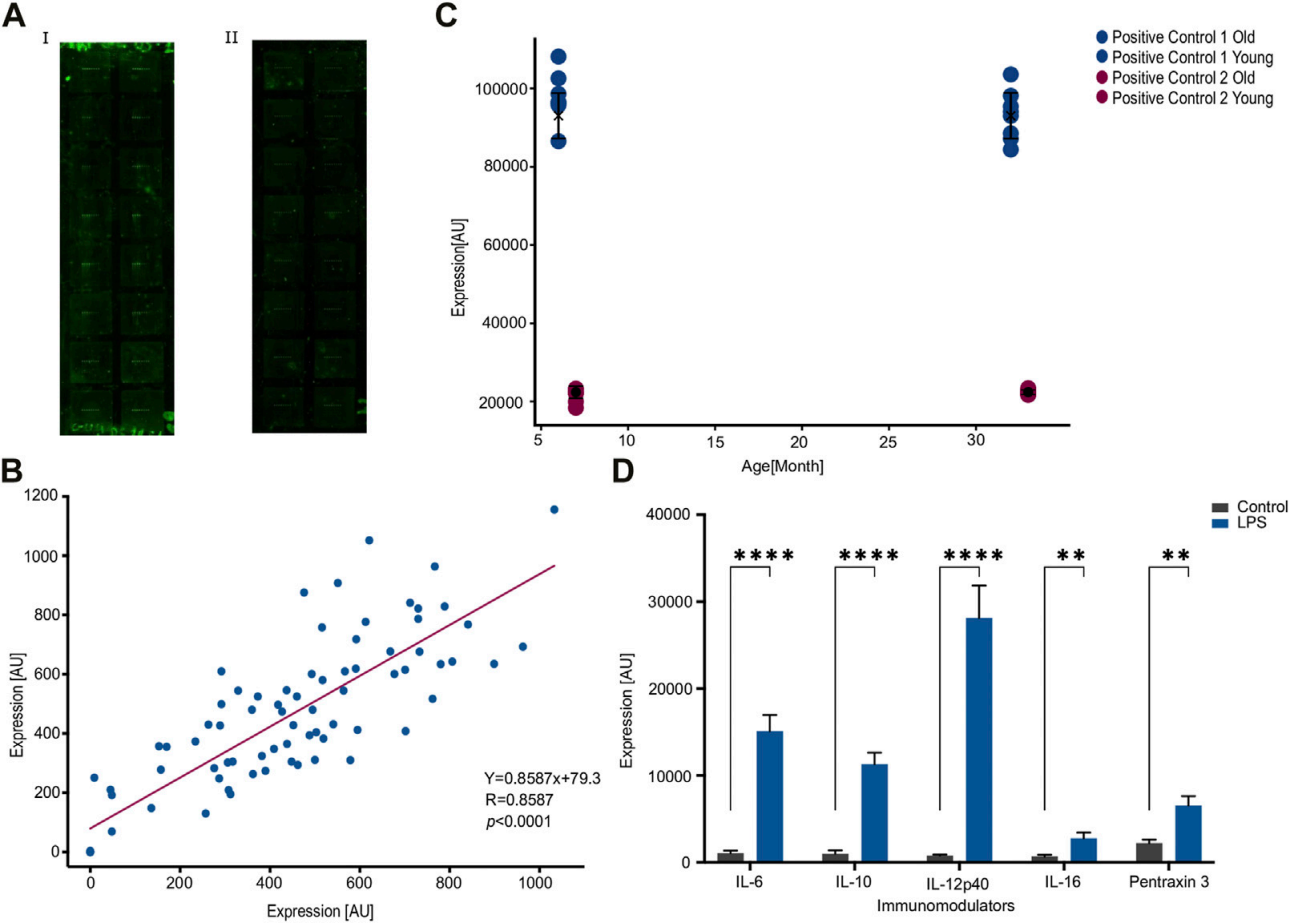
Consistency within an array following an optimized protocol. **(A)**. An improved washing protocol improves signal-to-noise ratio by reducing background noise. **(I)**. An image of the array using the manufacturer protocol. **(ii)**. An image of the array using the modified protocol. **(B)**. Correlation between duplicates across eight pairs of samples and nine features measured per sample, for a total of 72 measured values. Each dot represents a feature, where the x-and-y axes are the expression values from each sample of the corresponding duplicate. R = 0.858 under Pearson correlation (*p* < 0.0001) and more importantly the slope is 0.86. **(C)**. Internal positive controls are not affected by the type of samples. Two pairs of positive controls show no significant difference between the two age cohorts of 6 and 30 months of age. **(D)**. Stimulation by LPS increases the expression of published LPS-associated features. IL-6, IL-10, IL-12p40, IL-16, and Pentraxin 3 exhibit a significant increase due to LPS stimulation. Number of samples; *n* = 10. **p < 0.01, ****p < 0.0001. *p*-value was calculated using a Student’s t-test.

Figure 1AI illustrates a typical slide prior to the modifications implemented to the manufacturer’s protocol, whereas Figure 1AII illustrates a typical slide following the modifications. Slide 1b lower levels of background noise resulting in clearer profiles and improved accuracy of readings.

### Validating consistency within an array

In order to evaluate consistency within an array, we conducted a study using eight replicate samples in a 16-well device. Each well contained nine features, resulting in a total of 72 expression values. The results of this analysis are depicted in Figure 1B, where the expression of each feature in the duplicate samples is represented by a dot, and the values of each sample in the duplicate are plotted on the x- and *y*-axes.

The Figure shows a highly significant correlation between duplicates (R = 0.83, *p* = 6 × 10^(−22)^, Pearson correlation) with a slope of 0.86.

To further test the consistency within an array, we compared the values of control features that are intrinsic to each well. The eight replicates were composed of two groups of four animals each: one group consisted of 6-month-old hens and the other group comprised 30-month-old hens. The results, presented in Figure 1C, demonstrated that expression of control features were not significantly different between the two age cohorts (*p* = 0.8662 or 0.2546 for the two intrinsic controls, Figure 1C).

Next, we wanted to validate the quality of the array against a consensus treatment. To do this we injected the hens with LPS, a well-known stimulator of the immune system (Simpson and Trent, 2019), and compared the response to five established LPS-induced cytokines. In agreement with previous reports (Erickson and Banks, 2011; Magrini et al., 2016; Jia et al., 2021), stimulation by LPS increased the expression significantly in the already established LPS-related cytokines and Pentraxin 3 (Figure 1D). Taken together, the significant correlation between duplicates within an array and the expected response to LPS indicate a specific recognition of the antibodies against their corresponding targets.

### Validating consistency between arrays

One important aspect of scientific studies is reproducibility between similar experiments. We, therefore, sought to evaluate the consistency between arrays by performing two pairs of repeats comparing young and old animals. We then evaluated the consistency between the arrays by calculating the correlations between the readings of the same features in both arrays.

We chose to focus on an aging-related signal for several reasons: a) our lab studies aging and we wanted to confirm the ability of the array to detect changes in cytokine expression associated with aging; b) the use of LPS as a positive control does not reflect a typical biological process, as LPS is an artificial and potent immune stimulator; and c) the immune system undergoes senescence with age (Nikolich-Žugich, 2018), providing a natural biological context for evaluating changes in cytokine expression and testing the array.

**FIGURE 2:**
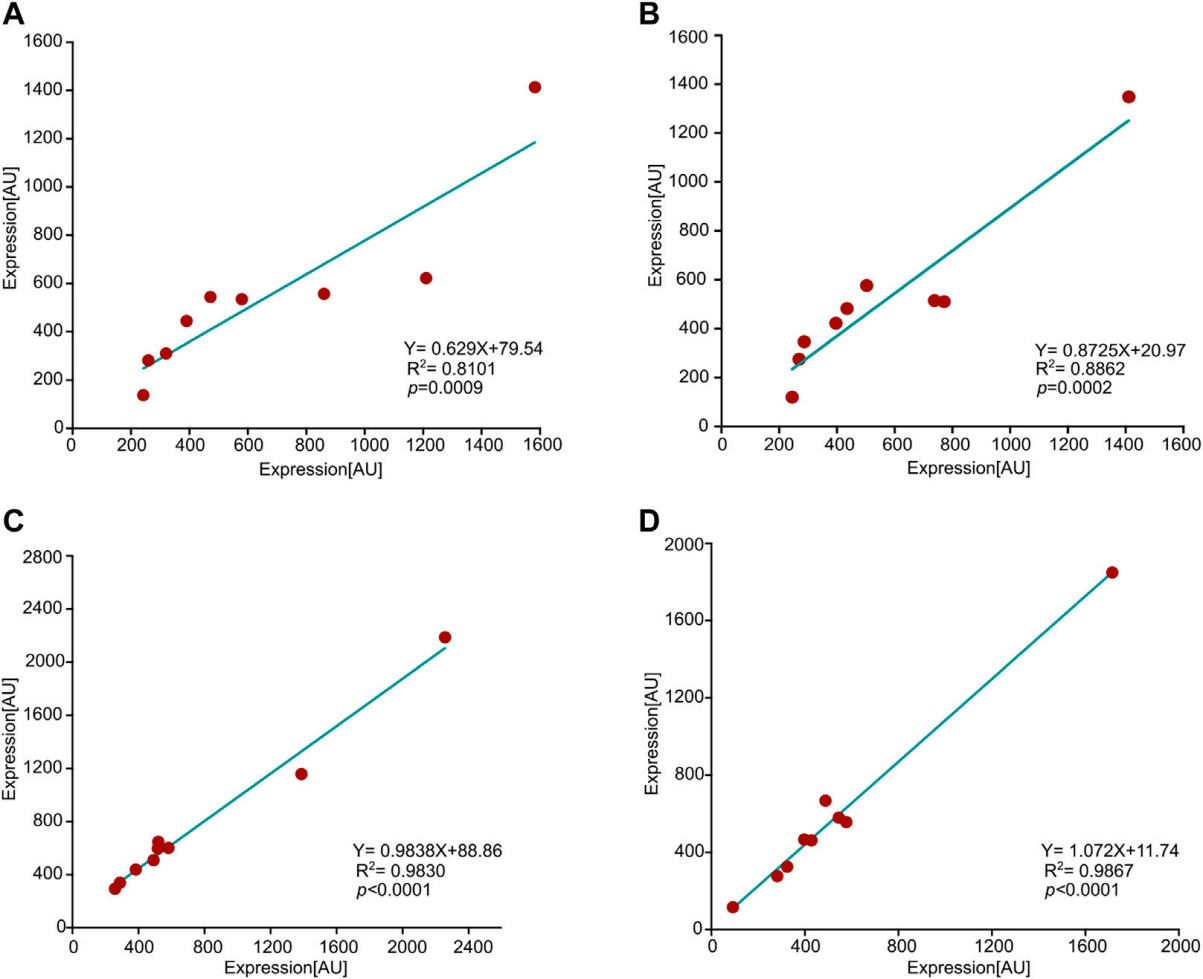
Consistency between arrays. Shown are correlations between four pairs of arrays, each pair is associated with the same age. Each dot represents a feature, and the x-and-y axes represent the expression values of that feature in the corresponding array. The values of each feature reflect the mean over eight animals. *R* ^2^, *p*-value (Pearson), and slopes are all presented in the graphs. **(A)** 9 months old hens, **(B)** 29 months, **(C)** 12 months, and, **(D)** 26 months.

Figure 2 illustrates the correlation between expression values in two pairs of arrays comparing young-vs-old hens; one pair corresponds to 12-vs-26 months of age, and the other to 9-vs-29 months of age. Each array contains 16 samples, eight of each age group, and the expression of each feature represents the mean value calculated for eight hens. All four age groups demonstrated a very significant correlation between features corresponding to animals of the same age. Thus, similarly to consistency in duplications within an array, there is consistency between arrays.

Last, we assessed the array under a biologically relevant context by identifying age-related changes in cytokine expression. Since previous research has not found a consistent pattern in cytokine expression with age, including in humans (Kim et al., 2011; Minciullo et al., 2015), we utilized the median value of all features to represent the systemic behavior of the cytokines as the animals senesce. The value for each cytokine reflects the average of eight animals in its age group.

**FIGURE 3:**
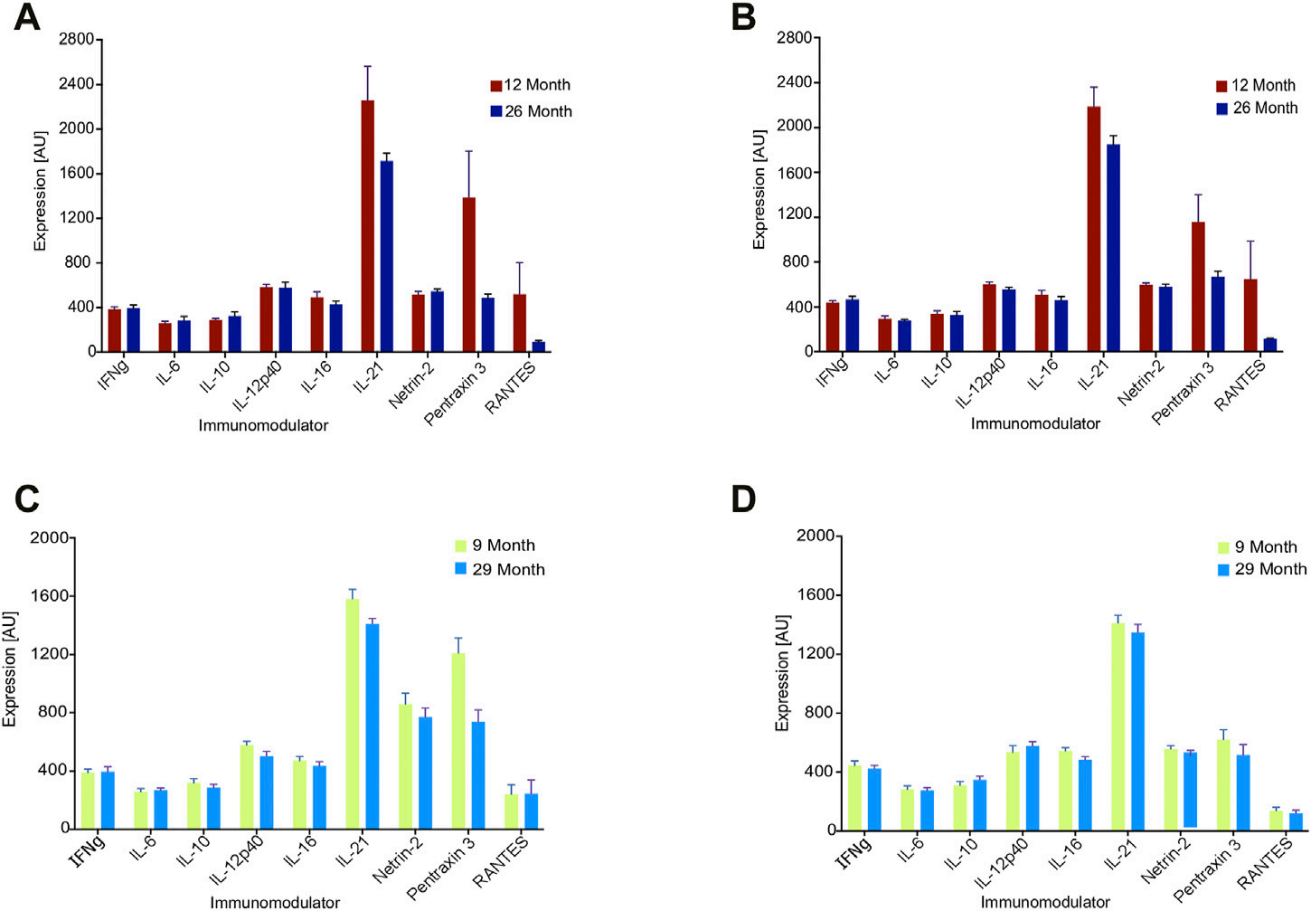
Expression values of nine features of the array for the denoted age cohorts. Each value reflects an average over eight hens. **(A, B)** 12 vs. 26 months of age, **(C, D)** 9 vs. 29 months of age.

Figure 3 illustrates the impact of aging on the four arrays from Figure 2, with one pair consisting of 9-vs-29 months arrays and the other pair consisting of 12-vs-26 months arrays. In all arrays, the median value of the features decreased with age: The 9-vs-29-month-old set exhibited old-to-young median ratios of 0.9 and 0.92, while the 12-to-26-month-old set had old-to-young median ratios of 0.83 and 0.78 (Figure 3). Significance for the four old-to-young ratios was calculated using random permutations of the animals to have *p* = 0.0001. The explicit values of each cytokine are presented in Figure 3: while the behavior of individual features may not change significantly with age, the collective behavior is very consistent.

We also calculated the old-to-young median ratio for the 6-vs-30-month array from above and found it to be 0.7. However, because hens at 6 months of age are still before peak egg production and may not be considered fully functional adults in the context of agriculture, we chose to focus on hens that are 9 months of age or older, which marks the onset of peak egg production.

In summary, the array consistently demonstrated that aging is associated with an overall decline in cytokine expression.

## Discussion

Chicken eggs constitute one of the most affordable sources of animal protein available (Bain et al., 2016). One key factor in maintaining high egg production rate is a fully functional immune system that can defend against the various pathogenic challenges in the chicken facilities. Additionally and unlike laboratory model animals, the immune system of hens is constantly exposed to various challenges of the complex housing environment of chicken facilities, including pathogens and stress (Bendikov-Bar et al., 2021). This makes the laying hen a valuable model for studying immunology in a realistic setting that is similar to humans.

Despite the importance of laying hens to our nutrition, economy and being a valuable immunological model, there are insufficient standard and commercially available tools for studying avian immunology compared to their mammalian counterparts. Today, the commercially available repertoire of avian cytokines consists of about 25 elements, all of which were utilized by ELISA or Western blotting, and the list is still expanding (Lu et al., 2023). For example, a recent study has calibrated an ELISA assay for chicken IL-2, IL-6, IL-10, IL-12p40, and IFN-γ (Krzysica et al., 2022). The antibodies reliably detected native cytokines and exhibited sensitivity as low as 32 pg/mL. For example, a chicken multiplex cytokine assay (Bio-Plex) quantified multiple chicken cytokines following a challenge with Rous sarcoma virus (RSV) for immunized and control animals (Mucksová et al., 2018).

This study introduced a commercially available cytokine antibody array as a reliable tool to measure the expression of circulating cytokines and immune modulators in laying hens. One confining factor in achieving reproducible results was a relatively low signal-to-noise ratio due to high background noise. We, therefore, modified the manufacturer protocol to reduce background noise and improve the sensitivity of the array (Figure 1).

We then validated the activity of the antibodies against a potent immune stimulator in LPS as a positive control and assayed the consistency of the array both intrinsically and extrinsically for unstimulated animals. Specifically, we found a very significant correlation between duplicates within an array and reproducibility between arrays among biological repeats. Because LPS is such a strong immune stimulator and does not reflect a typical biological process, we decided to test for consistency in age-related differences in expression. Indeed, there was a good correlation between different arrays upon running the same age groups (Figure 2).

On the biological front, the array demonstrated a consistent decrease in overall cytokine expression with age across five separate repeats. One way to interpret this result is that in old hens the immune system becomes inefficient, which lowers its ability to respond to immune challenges. Aging, therefore, lowers the dynamic range of immune activity, which results also in lowering the median expression of the features in the array.

One additional aspect that was assayed by assessing repeatability was the small sample size needed to distinguish between cohorts. We found that eight birds were sufficient to observe age-related differences in overall cytokine expression.

Satisfying with eight animals for consistency is a result of two reasons: First, as opposed to ‘Omics’ data, there is no need to correct for multiple hypotheses (Benjamini and Hochberg, 1995). Namely, we use the directly calculated *p*-value without the need to compensate for multiple hypotheses and adjust the calculated value accordingly. Note that in ‘Omics’ experiments, the relevant features that are significantly different between cohorts are not known, and one has to screen through many irrelevant features, therefore generating a factor of multiple hypotheses (Benjamini and Hochberg, 1995). To compensate for this factor one typically needs to increase the number of samples, which improves the separation between the means of the cohorts resulting in a better *p*-value. In the cytokine array, however, the relevant features were already selected, because the role of each feature in the immune system is already known, and no correction is needed. Second, using a small number of animals suggests that the array is sensitive to differences between cohorts, more than to the variance within each cohort. Separating between cohorts is a combination of population variance with the quality of the array in terms of signal-to-noise ratio.

The reproducible decline in cytokine expression with age using eight animals per group further supports the validity of the array as a tool for assessing avian immunology.

## Data Availability

The original contributions presented in the study are included in the article/Supplementary material, further inquiries can be directed to the corresponding author.
